# Accuracy of an XGBoost-based privacy preserving record linkage system compared with an electronic health record patient matching module in identifying patients shared between nearby academic health centers

**DOI:** 10.1093/jamia/ocag020

**Published:** 2026-03-19

**Authors:** Douglas S Bell, Tawny Saleh, Fernando Javier Sanz Vidorreta, Cenan N Pirani, Joshua M Pevnick, Robert A Jenders, Spencer L Soohoo

**Affiliations:** Department of Medicine, David Geffen School of Medicine, University of California, Los Angeles, CA 90095, United States; Clinical and Translational Science Institute, University of California, Los Angeles, Los Angeles, CA 90095, United States; Division of Preventive Medicine, David Geffen School of Medicine at UCLA, Los Angeles, CA 90095, United States; Division of Informatics, Department of Computational Biomedicine, Cedars-Sinai Health System, Los Angeles, Los Angeles, CA 90048, United States; Clinical and Translational Science Institute, University of California, Los Angeles, Los Angeles, CA 90095, United States; Clinical and Translational Science Institute, University of California, Los Angeles, Los Angeles, CA 90095, United States; Clinical and Translational Science Institute, University of California, Los Angeles, Los Angeles, CA 90095, United States; Department of Medicine, Cedars-Sinai Medical Center, Los Angeles, CA 90048, United States; Department of Medicine, David Geffen School of Medicine, University of California, Los Angeles, CA 90095, United States; Clinical and Translational Science Institute, University of California, Los Angeles, Los Angeles, CA 90095, United States; Charles R. Drew University of Medicine and Science, Los Angeles, CA 90059, United States; Clinical and Translational Science Institute, University of California, Los Angeles, Los Angeles, CA 90095, United States; Research Informatics and Scientific Computing Core, Cedars-Sinai Medical Center, Los Angeles, CA 90048, United States

**Keywords:** medical record linkage/methods, electronic health records/standards, health information exchange/standards

## Abstract

**Objectives:**

Patients often receive health care from multiple organizations. Privacy Preserving Record Linkage (PPRL) is a technology for linking patient records without releasing personally identifiable information. We compared a commercial PPRL tool that uses the XGBoost machine learning algorithm with Care Everywhere (CE), a widely used rule-based patient linkage module.

**Materials and Methods:**

We matched the complete patient populations from Cedars-Sinai Health System and University of California, Los Angeles (UCLA) Health using the XGBoost PPRL tool at each of 3 score thresholds (98, 95, and 90), reflecting stricter vs more permissive matching. We compared PPRL matches with CE matches for the cohort of 849 157 patients who had been queried by CE from UCLA to Cedars-Sinai over 18 months. To classify proposed matches as false, uncertain or correct matches, 2 reviewers manually reviewed a random sample of 1200 patients representing each category of matches.

**Results:**

Care Everywhere matched 18% of the cohort, whereas PPRL matched 9%, 27%, and 29% of the cohort using the 98, 95, and 90 thresholds, respectively. Projecting the false match rates from the manual review to the original populations, precision for CE was 99.6% (95% CI, 97.8%-100%). Precision for PPRL was 100% (95% CI, 99.2%-100%), 99.4% (95% CI, 97.4%-99.9%), and 98.7% (95% CI, 96.5%-99.4%) at the 3 thresholds, respectively. Using CE and PPRL matches together as a proxy gold standard, recall for CE was 61.5% (95% CI, 60.3%-61.9%) and for PPRL was 30.6% (95% CI, 30.3%-30.7%), 92.2% (95% CI, 90.2%-92.7%), and 96.8% (95% CI, 94.6%-97.5%) at each threshold, respectively.

**Conclusions:**

The precision and recall of PPRL matching differed substantially across the available match thresholds. Compared with the rule-based system, PPRL at the 95 threshold had 50% higher recall with similar precision. Privacy Preserving Record Linkage holds promise for improving research, but users must choose the precision vs recall needed for their application.

## Background and significance

Many patients receive health care from multiple institutions, leading to important data on observations and treatments being missing from the records at any 1 institution.^1^ This fragmentation can result in unsafe or misinformed patient care, impede quality assurance, and lead to errors in research using real world data, either for epidemiology or for clinical trial recruitment. To address this challenge for patient care, some electronic health record (EHR) systems provide for cross-institutional patient matching and data exchange before each patient visit. Epic, the EHR system used at both institutions in the present study, developed a data linkage module called Care Everywhere (CE) to match patients across institutions based on personally identifiable information (PII) such as name, address, telephone number, and dates of birth.^2^ Once patients are matched, one EHR can retrieve information from the other and display it to health-care providers at the time they are actively caring for a patient. We previously found CE linkages to be highly accurate.^3^ However, CE requires the comparison of PII from the patient records at each end of the match.

Privacy Preserving Record Linkage (PPRL) is a different approach to link patients across institutions, which does not require any release of PII.^4^^,^^5^ This approach uses cryptographic hashing to transform PII features into encoded values that can be compared without using the underlying values. First, each originating institution runs tokenizer software that creates an array of PII feature combinations (for example, “last name [LN] +first initial of first name [FN] + gender + date-of-birth [DOB]”) and then encodes each using 1-way hashing such that the original identifying information cannot be recovered. The array of these tokens is then further encrypted for secure transport to a third party that will conduct matching. The matching party then compares the arrays of tokenized values from each originating institution to determine potential matches, with no use of the original personal information.

Privacy Preserving Record Linkage is being adopted increasingly to link EHR data. In an early effort, a PPRL algorithm was used to match patients between a clinical study dataset and academic health-care medical records with a sensitivity of 96% and specificity of 100%.^6^ A recent systematic review identified 2 additional PPRL evaluations that used EHR data.^7^ One of these used a self-developed PPRL tool to link EHR data with insurance claims.^8^ The other used a commercial PPRL system on a sample of 20 000 potential duplicate patients from a single institution.^9^ We are not aware of prior studies that have evaluated the use of PPRL in the setting of actual patient data exchange as implemented between EHRs from 2 distinct health-care institutions.

We sought to compare links made by a commercial PPRL system that makes matches using an XGBoost machine learning algorithm vs links that were actually made by Epic’s rule-based CE module, between 2 neighboring provider organizations, University of California, Los Angeles (UCLA) Health and Cedars-Sinai Health System. The institutions’ primary hospitals are located 5 miles apart, and patients commonly receive care at both organizations. The commercial PPRL system has undergone an “Expert Determination,” showing that it meets federal standards for privacy.^10^ The commercial PPRL system’s matching can be implemented at different levels of stringency by varying the XGBoost score threshold used to consider a record pair a match. We sought to evaluate the system’s recall and precision of at each of 3 suggested thresholds, compared with the CE patient matching system.

## Methods

### Study setting

This study compared the accuracy of Datavant’s Match PPRL system with the patient matching that was routinely conducted for patient care using Epic’s CE module between UCLA Health and Cedars-Sinai Health System over 18 months (January 1, 2021-June 30, 2023). As we previously described, the evening before a scheduled patient encounter, the Epic CE module automatically queries nearby participating Epic institutions for records from the same patient. We previously described the CE algorithm in detail,^3^ but in brief it uses rule-based deterministic matching to compare 12 identifiers including exact name, soundex(name), exact date of birth, year of birth, exact email address and exact phone, with different weights assigned when each type of identifiers match. (Organizations can fine-tune the algorithm by adjusting these weights, but both UCLA and Cedars-Sinai use the default CE settings, and neither uses Social Security Numbers.) A “successful” link is established if the sum of weights for the potential match exceeds a predefined threshold and is unique. Otherwise, the query is considered “unsuccessful.” Once a successful link is established, the patient is no longer queried between the same institutions.

### PPRL matching

Both UCLA and Cedars-Sinai independently implemented the Datavant PPRL tokenization software locally. The software generated hashed tokens representing 18 PII feature combinations (Table S1). Each institution tokenized all patients in their respective EHR systems (6.0 million patients at UCLA and 5.2 million patients at Cedars-Sinai) so that the Datavant match process would have the same opportunity as CE to match any patient. Preprocessing of the identifiers was limited to removal of hyphens from SSNs and phone numbers and standardization of birth dates to “mm/dd/yyyy” format.

**Figure 1.**
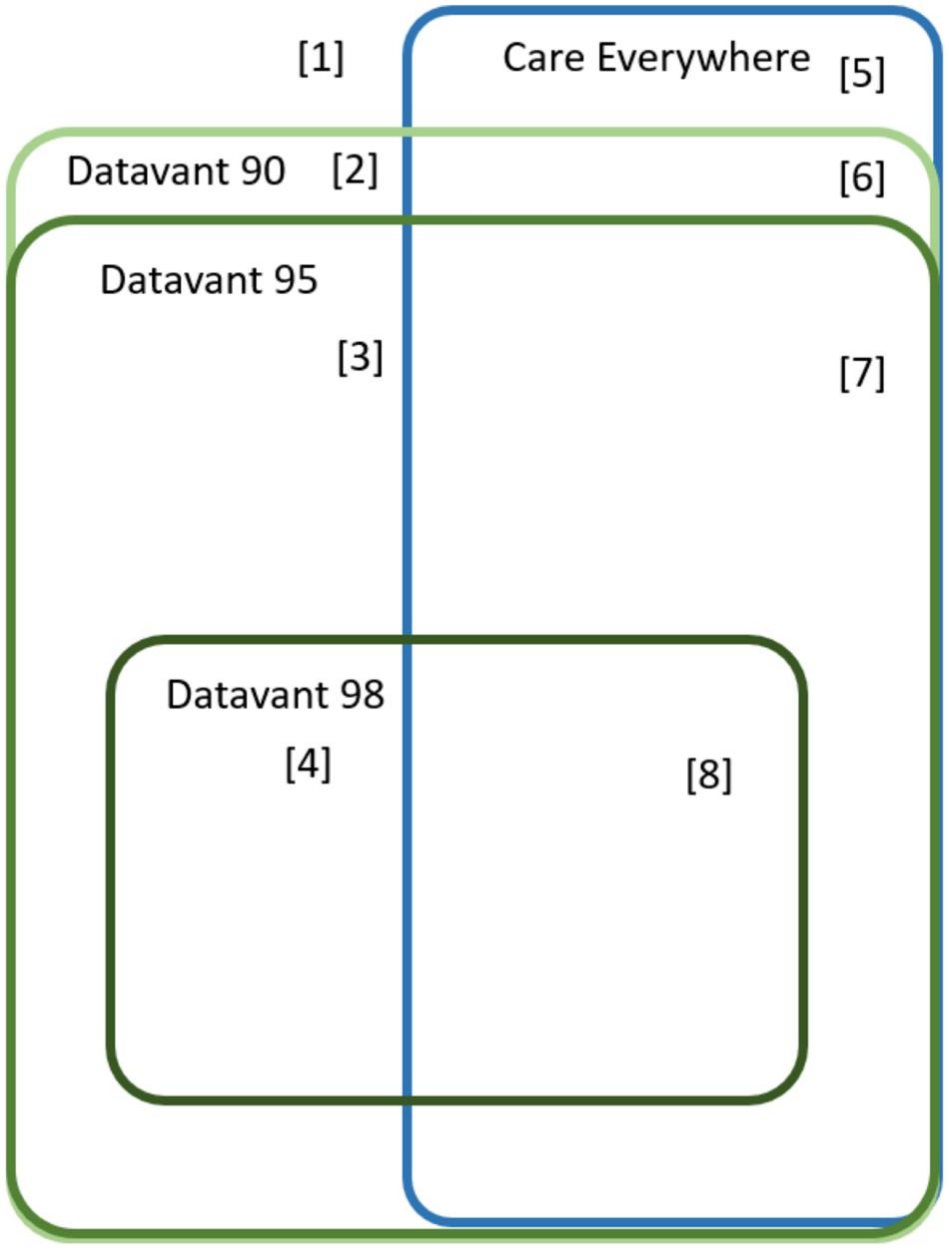
Euler diagram showing overlap of the match algorithms. Schematic showing containment of the 3 Datavant (PPRL) match algorithms and their overlap with CE matches. The combination results in 8 mutually exclusive match categories, with each category shown as a numbers in brackets. These numbers, for example ‘[1]’, correspond to the row numbers for each exclusive category in Table 2.

Tokenized files from each institution were then uploaded to Datavant for them to act as the “Linkage Honest Broker” for this project. Datavant applied its “Match” algorithm, which uses a set of pretrained XGBoost machine learning models to calculate a score reflecting the Jaccard distance^11^ between potential matches. Match scores are adjusted using a set of precision-recall curves derived by running the model on multiple benchmarking datasets. This process results in a score from 0 to 100 for each potential match, reflecting the model’s prediction of the match probability. The threshold used to consider a potential match as successful can be set at different scores depending on the use case. For this study, we used the 3 score thresholds, 90, 95, and 98 that Datavant recommends to optimize both precision and recall. As shown in Figure 1, patients who match at the strictest threshold (98) are a subset of those who match at the next threshold (95) and they are a subset of those who match at the most permissive threshold (90).

Based on these thresholds, each patient’s PPRL match status fell into one of the 4 mutually exclusive categories: no match at any threshold, matching only at the Datavant 90 threshold, matching at the Datavant 90 and 95 thresholds, or matching at all 3 Datavant thresholds, 90, 95, and 98.

### Comparison of PPRL with care everywhere match results

We compared PPRL matches with CE match results for all patients who had been queried by CE from UCLA to Cedars-Sinai for possible patient linkage from January 1, 2021 to June 30, 2023. For each of these patients, we retrieved data on the outcome of CE queries using the UCLA Epic Systems’ Clarity database. Patients with a successful CE link were classified as CE matches, while those with no successful link were categorized as nonmatches. Combining these 2 CE categories with the 4 Datavant match categories described above created 8 mutually exclusive match categories for each patient (as shown in Figure 1). Two examples are patients who did not match with CE and who matched with all 3 Datavant thresholds (Figure 1, category 4), and patients who did match with CE and who matched with Datavant only at the 90 threshold (Figure 1, category 6).

### Manual review and precision analysis

We manually reviewed simple random samples of 200 patients from 6 of the 8 possible CE×Datavant match categories. We excluded the 2 categories in which CE and all 3 Datavant algorithms were in full agreement, assuming that these are all correct matches or nonmatches. We chose the sample size of 200 because it would give a 95% CI of 1%-6% assuming an average error of 3%.

For each sampled patient, we separately retrieved demographic data from the UCLA and Cedars-Sinai EHRs including first and last names, dates of birth, and addresses, their encounter date range, and, when available, each patient’s email address, height, weight, body mass index, and social security number (SSN). We also cross-referenced each patient’s first and last names to ranked lists of the 100 most common first names^12^ and last names^13^ in the United States. Using defined criteria (Table S2), 2 independent reviewers (T.S. and D.S.B.) classified each patient pair as a true match (the same person), false match (FM) (not the same person), or uncertain match. These criteria included searching an online index such as whitepages.com for the existence of a single person having the differing names and/or contact information from each side (confirming a match) or for the existence of separate people having the differing names and/or contact information from each side (confirming an FM). Discrepancies between reviewers were resolved through discussion and consensus.

To estimate the precision of CE and each the 3 PPRL algorithms, we first calculated an FM rate for each sample, which was defined as the number of FMs divided by the total number of reviewed cases minus the number of uncertain matches (essentially, excluding the uncertain matches from the FM rate denominator). We then calculated a 95% CI for each FM rate estimate using a binomial (exact) calculation for each proportion. Next, we projected these rates to the full population within each category by multiplying the category count by the FM rate estimate as well as by the lower and upper bounds of its 95% CI. These category-specific FM projections were then summed for the categories that make up each algorithm and divided by the total patient matches for each algorithm according to the formula Precision_a_=(All matches_a_−False matches_a_)/All matches_a_, where “a” represents each algorithm. This is equivalent to true positives/(true positives+false positives).

### Recall analysis

To estimate the recall of CE and each of the 3 PPRL algorithms, we assumed that matching on either the CE or the permissive PPRL algorithm constituted a potential match that was either found or not found by the given algorithm, making the combination substitute for a gold standard in the recall analysis. The recall rate for each algorithm (*a*) was then calculated according to formula Recall_a_=(All Matches_a_−False matches_a_)/Matches_any_. This is equivalent to true positives/(true positives+false negatives). We calculated a 95% CI for each estimate using the 95% upper and lower bounds of the recall proportion (using the normal approximation of the binomial) plus or minus, respectively, the 95% upper and lower bounds of the projected FM count from the precision estimates.

All aspects of the study were reviewed and approved by the UCLA and the Cedars-Sinai Medical Center Institutional Review Boards (IRBs). Neither Datavant nor Epic was involved in the study design or the analysis.

## Results

### Initial results for each exclusive match category

**Figure 2.**
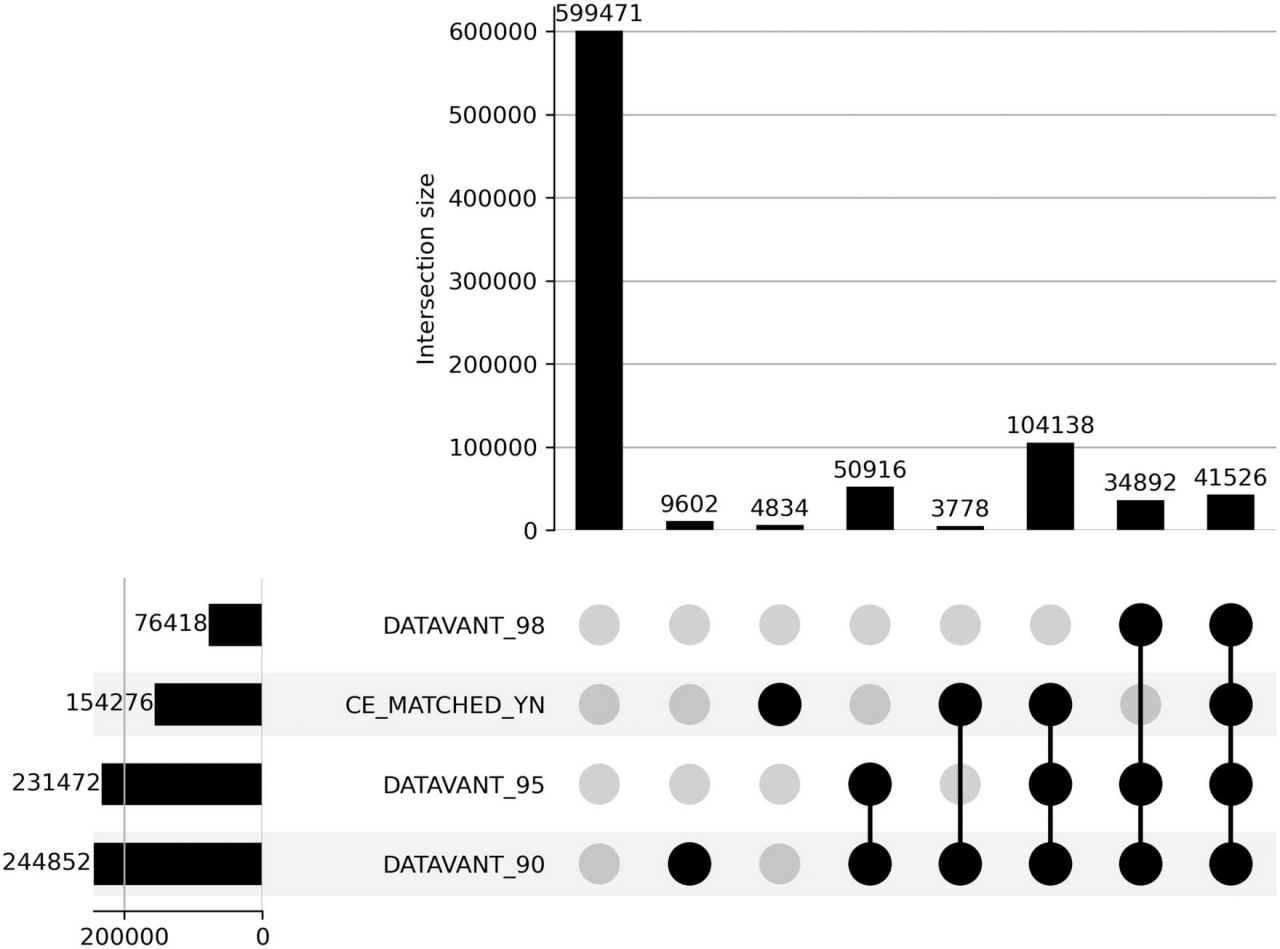
UpSet plot showing match results for each match category and each algorithm.

University of California, Los Angeles sent 849 157 CE queries to Cedars-Sinai during the 18-month study period. Within this base population, each patient either matched or did not match with CE, and each patient’s PPRL match results fell into one of the 4 possible categories described in the “Methods” section, based on the match threshold. The left columns of Table 1 show that the base population included a diversity of patient ages, genders, ethnicities, and races. Table 2 shows each patient’s CE match and PPRL match, forming the 8 mutually exclusive categories shown with each patient represented once. Figure 2 is an UpSet plot^14^^,^^15^ displaying visually how the base population of patients was distributed among the algorithms and the exclusive categories. Among the 95 410 matches that were found only by the PPRL system and not CE, 37% were identified using the Datavant 98 algorithm, an additional 53% were identified using the Datavant 95 algorithm, and an additional 10% were identified using the Datavant 90 algorithm (rows 2-4 of Table 2).

**Table 1.** Characteristics of the base population vs the manual review sample.

Variable	Value	Base population (*n*=849 158)		Manual review sample (*n*=1200)	
		Count	Percentage	Count	Percentage
Age (years)	0-10	60 355	7.1	42	3.5
	11-20	80 162	9.4	62	5.2
	21-30	104 310	12.3	131	10.9
	31-40	139 312	16.4	195	16.3
	41-50	116 961	13.8	148	12.3
	51-60	109 563	12.9	181	15.1
	61-70	108 471	12.8	208	17.3
	71-80	77 874	9.2	133	11.1
	81-90	37 042	4.4	51	4.3
	91-100	11 462	1.3	25	2.1
	101-110	1087	0.1	–	
	Missing or invalid	2559	0.3	24	2.0
Gender	Female	455 503	53.6	657	54.7
	Male	389 276	45.8	515	42.9
	Missing or invalid	4379	0.5	28	2.3
Ethnicity	Hispanic	155 708	18.3	171	14.2
	Not Hispanic	480 462	56.6	686	57.2
	Choose not to answer	96 537	11.4	149	12.4
	Unknown	113 892	13.4	175	14.6
	Missing or invalid	2559	0.3	19	1.6
Race	American Indian or Alaska Native	3675	0.4	4	0.3
	Asian	88 462	10.4	108	9.0
	Black or African American	40 671	4.8	71	5.9
	Middle Eastern or North African	16 722	2.0	25	2.1
	Native Hawaiian or other Pacific Islander	2094	0.2	2	0.2
	Other	126 907	14.9	163	13.6
	White or Caucasian	268 695	31.6	401	33.4
	Multiple races	36 424	4.3	51	4.2
	Do not identify with race	28 516	3.4	33	2.7
	Patient refused	91 855	10.8	129	10.7
	Unknown	121 766	14.3	163	13.6
	Missing or invalid	23 371	2.8	51	4.3

**Table 2.** Total matches in each exclusive category of Care Everywhere match and PPRL match.

CE match	Datavant PPRL match	Row ID	*n*, cell (%)
No match	No match	1	599 471, 70.6
	90 only	2	9602, 1.1
	90 and 95	3	50 916, 6.0
	90, 95, and 98	4	34 892, 4.1
Match	No match	5	4834, 0.6
	90 only	6	3778, 0.4
	90 and 95	7	104 138, 12.3
	90, 95, and 98	8	41 526, 4.9
Total			849 157, 100.0

Abbreviation: PPRL, Privacy Preserving Record Linkage.

### FM rate and precision estimates

The right columns of Table 1 show the demographic characteristics of the simple random sample of 1200 UCLA and Cedars-Sinai patient pairs (200 from each of 6 match categories) taken for chart review. As expected, the random sample had similar characteristics except that Hispanic patients and children were less represented in the sample. The sample had good representation of children, elders, women, and racial/ethnic minorities.

In the manual review, 27 of the sampled patients were excluded due to their EHRs being “restricted,” leaving 1173 patients whose data from each institution was analyzed. Among the 1173 pairs, 683 had identical first names, 874 had identical last names, all had identical dates of birth and 503 had a first or last name among the 100 most common first or last names in the United States. Table 3 shows the counts of patient matches within each sampled category that met criteria for being false or uncertain matches. Reasons for FMs included patients with similar common names who happened to share a date of birth, and patients who have similar names and common dates of birth due to being twins. Reasons for uncertain matches included missing data from one side or the other and an inability to verify demographics from one side or the other in an online identity service.

**Table 3.** Category-specific manual review results, net false match (FM) rate estimates, 95% CI, and projections to the base population.

CE and Datavant PPRL category^a^	Sample reviewed	False match (FM) count n	Uncertain match (UM) count, *n*	FM rate denomin-ator^b^	Net FM rate estimate (%)	95% CI of FM rate^c^	FM rate projected to full category	Projected 95% CI
CE (N), only Datavant 90 (M)	200	30	35	165	18	13%-25%	1746	1248-2401
CE (N), Datavant 90 and 95 (M)	200	3	10	190	1.6	0.3%-4.5%	804	168-2291
CE (N), all 3 DV algorithms (M)	200	0	0	200	0	0.0%-1.8%	0	0-628
CE (M), all 3 DV algorithms (N)	176	1	1	175	0.6	0.6%-3.1%	28	29-149
CE (M), only Datavant 90 (M)	200	3	3	197	1.5	0.2%-4.4%	58	6.0-166
CE (M), Datavant 90 and 95 (M)	197	1	9	188	0.5	0.0%-2.9%	554	10-3020

a (M)=the patient pair matched with the indicated algorithm; (N)=the patient pair did not match with the indicated algorithm.

b *n* reviewed minus *n* uncertain.

c Estimated using the binomial exact distribution of the net false match (FM) rate proportion.

Table 3 also shows the category-specific FM rate denominators and net FM rates with 95% CIs as well as the projection of these rates and CIs to the original populations. Table 4 shows that after summing projected FM counts and 95% CIs of the categories that make up each algorithm, CE showed a precision of 99.6% (with 95% CI, 97.8%-100%). The Datavant-98 (strict), -95 (balanced), and -90 (permissive) algorithms showed precisions of 100%, 99.4%, and 98.7%, respectively (with the 95% CIs shown).

**Table 4.** Net precision and recall estimates for each algorithm.

Match algorithm	Table 2 **rows summed**	Total matches	Projected errors (95% bounds)	Precision (%) (95% CI)	Total−false matches (95% bounds)	Recall (%) (95% CI)
Care Everywhere	5-8	154 276	639 (54, 3336)	99.6 (97.8%-100%)	153 637 (150 652, 154 524)	61.5 (60.3%-61.9%)
Datavant 98 (strict)	4,8	76 418	0 (0, 628)	100 (99.2%-100%)	76 418 (75 652, 76 556)	30.6 (30.3%-30.7%)
Datavant 95 (balanced)	3,4,7,8	231 472	1358 (178. 5939)	99.4 (97.4%-99.9%)	230 114 (225 298, 231 529)	92.2 (90.2%-92.7%)
Datavant 90 (permissive)	2-4,6-8	244 852	3161 (1443, 8506)	98.7 (96.5%-99.4%)	241 691 (236 215, 243 551)	96.8 (94.6%-97.5%)

### Missed match rates and recall

Defining potential matches as those patients who matched using either CE or the Datavant-90 algorithm (equivalent to any match, or rows 2-8 in Table 2), there were 249 686 potential matches (29.4% of the base population). As shown in Table 4, last columns, among these potential matches, after subtracting projected erroneous matches from total matches, the CE algorithm had a recall rate of 61.5% (95% CI, 60.3%-61.9%). In the manual review, the most common reason for CE having missed matches that PPRL had found was due to the PPRL algorithm’s use of SSN in its matching. Other reasons included first or last name misspellings or typos, differences in the inclusion of middle names or initials in first names, differences in compound first or last names (eg, Smith vs Smith-Jones), and differences in phone numbers, emails and/or addresses.

Analogously, Datavant PPRL at the 98 threshold had a recall of 30.6%, at the 95 threshold had a recall of 92.2%, and at the 90 threshold had a recall of 96.8% (with the 95% CIs shown in Table 4). Of note, 4834 (1.9%) of the 249 686 potential matches were matched only by CE and not by PPRL. In the manual review, the most common reason for PPRL having missed matches that CE had found was due to differences in compound first or last names; other reasons were similar to the reasons that CE missed matches.

## Discussion

Health information exchange (HIE) has created substantial value for patient care delivery by decreasing redundant testing and by preventing admissions from the emergency room, among other effects.^16–18^ We previously found that Epic’s CE HIE system produced accurate and reasonably complete patient linkages across neighboring health systems.^3^ However, HIE methods typically link patients based on exchanging their identifying characteristics, which creates governance challenges for their use in large-scale research. The method of PPRL^4^^,^^7^ may make health-care organizations more willing to participate in multisite networks and is gaining adoption for patient-oriented research as a means to merge fragmented records and link EHR data to external sources such as claims data^19^ and clinical trial data.^20^ In a large-scale implementation within the National Clinical Cohort Collaborative (N3C), an earlier, rule-based version of the Datavant PPRL system was used to link 9.5M patient records from 35 sites.^21^ This effort identified 135 037 patients having records from more than 1 institution (1.4%) and 68 536 patients having duplicate records within the contributing institution (0.7%). The N3C study did not assess the accuracy or completeness of these matches, but the N3C initiative is continuing to expand its use of PPRL.^22^ Another recent review highlights the potential of PPRL to improve the linkage of public health data, such as COVID-19 case and vaccination data.^23^

In this study, we assessed the precision and recall of a commercial PPRL system that uses the XGBoost machine learning algorithm, within a large cohort of patients whose linkage had already been assessed in practice using the CE implementation at our institutions. We found that CE matching in this population had precision of 99.6% and recall of 62%, which is consistent with the goal of avoiding erroneous EHR merges that could be difficult to undo. In comparison, the PPRL system’s performance differed substantially based on the match threshold selected. At the strictest threshold, precision was outstanding at 100% but recall was 31%, whereas at the most permissive threshold precision was still good at 98.7% but recall was much higher, 97%, using our gold standard substitute. At the balanced threshold (95), PPRL matching had precision of 99.4%, which is very similar to CE, but a recall of 92%, which substantially exceeds the recall of the CE matching that is currently in use. These findings show the importance of selecting thresholds for each record linkage application that prioritizes precision vs recall depending on the needs of the application.

In comparing the underlying reasons for the PPRL system’s having higher recall, the most common reason was its use of SSNs. We only evaluated the CE algorithm that was actually in use at our institutions. If circumstances had allowed for CE to include SSN in its algorithm, its recall would have been higher, but our institutions did not include SSN in the CE algorithm out of concern for patient privacy. The 1-way tokenization afforded by PPRL alleviated the privacy concern, enabling patients’ SSNs to be used in matching. Furthermore, the results overall show that the PPRL tokenization process did not degrade the matching algorithm’s performance. This shows that the hashed combinations of identifiers had not lost information compared with the unaltered identifiers.

We are aware of 3 prior studies that evaluated PPRL in matching EHR data. All of these prior studies used rule-based matching rather than a machine learning-based algorithm. One effort, using a precursor of the Datavant PPRL system, matched 2.3 million EHR identities vs 11 292 clinical study participants, 97% of whom were known to have a record in the targeted EHR.^6^ The researchers found 8 false positive and 477 false negative cases, which resulted in precision and recall rates of 99.9% and 95.7%. Another study used a locally developed rule-based PPRL tool to match EHR patients with insurance claims from Florida Medicaid.^8^ The investigators reported a precision of 97.3% among a random sample of 2511 patients whom they manually reviewed. They reported a recall of 75.5% among 1000 patients who were separately determined to match based on their Social Security and Medicaid ID numbers. The most recent study evaluated an earlier, rule-based version of the Datavant PPRL system (with 9 token combinations and 4 variations on the rule-based matching algorithm) in identifying duplicate patients within a random sample of 20 002 potential duplicates from a single health system’s EHRs.^9^ The researchers found precision rates ranging from 95% to 99.9% and recall rates ranging from 23% to 91%.

We were unable to find any prior study that used XGBoost in linking EHR data but we found 2 previous studies that used XGBoost in other record linkage applications. One study used an XGBoost model that had been trained on millions of record links created by individual contributors to a large, public, wiki-style family tree in order to link individuals across 100% samples of the US decennial censuses from 1900, 1910, and 1920.^24^ The authors report finding an overall match rate of 70% with a false positive rate of 12%, which at the time were considered “beyond the current frontier for record linking methods.” The other study linked CA vital statistics records to child protective services records based on names, dates of birth, and addresses from both parent and child, with the additional introduction of simulated errors.^25^ A novel XGBoost approach was compared with 2 previously tested commercial linkage solutions. Performance was similar for the 3 methods but the novel method automated several steps of the linkage including data cleaning and the selection of blocking. As in our study, results varied substantially based on the threshold selected to consider a record pair as matching.

Our study has several limitations. We involved only 2 institutions, each with its idiosyncratic processes for eliciting demographic information, within a specific region that has a diverse population but with characteristics that may differ from other areas of the nation. The manual review sample was limited to 1200 patients. A larger sample would have supported more precise estimates, but it is unlikely that more precise estimates would change the study conclusions. In estimating precision, we excluded uncertain matches from the error estimate denominator. If a majority of the uncertain matches are actually correct, our precision estimates are conservative but if a majority are incorrect then we are overestimating precision. In estimating recall, if we had evaluated a more permissive CE threshold, its recall would be higher, albeit at a lower precision. In both cases, the additional CE matches would raise the recall denominator for all of the algorithms, resulting in lower recall rates for PPRL. Our study did not evaluate any variations on the rule-based patient linkage algorithm that is in use by CE within our EHR systems. Had we been able to include SSNs and vary its point-based match threshold, CE’s performance might have been similar to the XGBoost system, albeit without privacy preservation.

Further research is needed to evaluate the accuracy of PPRL in linking EHRs from other communities and other health-care systems, given the variability we found. It is also important to periodically monitor the performance of previously evaluated record linkage implementations given the expected drift in local data. Privacy Preserving Record Linkage should also be evaluated in linking EHR data to non-EHR data sources such as insurance claims and registries such as the National Death Index.^26^ Research is also needed to examine the effect of using PPRL on research outcomes, for example in assessing the effect of obtaining more complete medication exposures for epidemiologic research or public health monitoring that uses “real world data.” However, future studies should note that because PPRL by design does not involve PII, studies of the PPRL precision would need IRB approval to obtain PII separately, as we did for this study.

Overall, our findings show that a commercial machine learning-based PPRL system can produce patient matches that are at least on par with a rule-based deterministic EHR-based patient matching system that is in widespread use. As importantly, our findings highlight the importance of selecting the appropriate match threshold based on the intended application. For clinical trial recruitment, a more permissive threshold may be desirable to identify and screen as many potential participants as possible. In contrast, for epidemiological research, a stricter threshold may be preferable to ensure a high degree of certainty when patient records are merged. Further research is needed on how to determine the best threshold for a given application when the data are hashed. Anecdotally, PPRL is also starting to be used in preparing datasets for public sharing,^27^ with tokens being generated and stored for future linkage with other data sources. As health systems increasingly participate in multicenter collaborations, integrating PPRL into data processing workflows is likely to take on greater importance.

## Conclusion

Privacy Preserving Record Linkage is capable of accurately linking the same patient’s data across different data sources without threatening their privacy, thus enhancing the accuracy of research, potentially on large scale. However, users must choose the balance of precision vs recall that is appropriate for their application. As researchers and health-care organizations seek to improve data interoperability and the efficiency of research, PPRL has the potential to play a crucial role in securely integrating fragmented patient records across diverse health systems.

## Supplementary Material

ocag020_Supplementary_Data

## Data Availability

The data on which this article is based cannot be made public due to its use of PII. De-identifying these data would make it meaningless.
