# Supplementary material for: Accuracy of an XGBoost-based privacy preserving record linkage system compared with an electronic health record patient matching module in identifying patients shared between nearby academic health centers
Source: J Am Med Inform Assoc. 2026 Mar 19;33(4):901–8. doi: 10.1093/jamia/ocag020 (PMC13089510; doi:10.1093/jamia/ocag020)
Supplement: ocag020_Supplementary_Data [file ocag020_supplementary_data.docx]

**Bell et al. 2025 Supplementary Tables**

Supplementary Table 1: PII variable combinations used to create Datavant tokens.

| last name + 1st initial of first name + gender + DOB  last name (soundex) + first name (soundex) + gender + DOB  last name + first name + DOB  last name + first name + DOB + zip3  last name + first name + DOB + zip5  last name + first name + gender + DOB  SSN + gender + DOB  SSN + first name  SSN + DOB  last name + email  first name + CellPhoneNumberUS | last name + 1st 3 characters of first name + gender + DOB + zip3  last name + 1st 3 characters of first name + gender + DOB  last name + 1st 3 characters of first name + gender + zip5  last name + first name + gender + zip5  last name + first name + gender + zip5 + birth year + birth month  last name + 1st initial of first name + DOB +zip3  last name (soundex) + first name (soundex) + DOB + Zip3 |
| --- | --- |

These 18 combinations of PII variables represent Datavant’s recommended tokens for healthcare organizations.

Supplementary Table 2: Manual Review Algorithm for Assigning Match Accuracy

| **Assignment** | **Criteria** |
| --- | --- |
| True Match | First Name (FN), Last Name (LN) & DOB match (accounting for misspellings, typos)  AND either  at least one other of cell phone, email, height, weight, SSN match (accounting for misspellings or typos)  OR only zip code matches plus rare First Name or Last Name  OR an online identity service* shows a single person having either  the differing contact information from each side matching the person’s online historical contact information  OR an uncommon name |
| False Match | The online identity service* shows two different people with the respective FN, LN, and DOB that are compatible with the different demographics from each side and no other fields match  OR the FN and LN are common, both sides have valid SSNs, and those SSNs differ  Examples:   - - Online sources* show matching twins who have similar names   - Online sources* show different people (living at the same time in different locations) with the same or similar FN & LN, the same DOB |
| Uncertain Match | The patient pair doesn’t match either the correct or erroneous criteria  Examples:   - - Common FN and LN, same zip, phones mismatch, other fields are missing on one side   - Rare FN, phone and zip differ, other fields blank on one side |

*: Including whitepages.com, fastpages.io, spokeo.com, truthfinder.com, 411.com
